# Estimated generic prices of cancer medicines deemed cost-ineffective in England: a cost estimation analysis

**DOI:** 10.1136/bmjopen-2016-011965

**Published:** 2017-01-20

**Authors:** Andrew Hill, Christopher Redd, Dzintars Gotham, Isabelle Erbacher, Jonathan Meldrum, Ryo Harada

**Affiliations:** 1 Department of Pharmacology and Therapeutics, University of Liverpool, Liverpool, UK; 2 Peninsula College of Medicine and Dentistry, Peninsula Medical School, Universities of Exeter and Plymouth, Plymouth, UK; 3 Faculty of Medicine, Imperial College London, London, UK; 4 Faculty of Medical Sciences, University College London, London, UK; 5 Department of Economics, University of Cambridge, Cambridge, UK

**Keywords:** CLINICAL PHARMACOLOGY, HEALTH ECONOMICS, ONCOLOGY, PUBLIC HEALTH, THERAPEUTICS

## Abstract

**Objectives:**

The aim of this study was to estimate lowest possible treatment costs for four novel cancer drugs, hypothesising that generic manufacturing could significantly reduce treatment costs.

**Setting:**

This research was carried out in a non-clinical research setting using secondary data.

**Participants:**

There were no human participants in the study. Four drugs were selected for the study: bortezomib, dasatinib, everolimus and gefitinib. These medications were selected according to their clinical importance, novel pharmaceutical actions and the availability of generic price data.

**Primary and secondary outcome measures:**

Target costs for treatment were to be generated for each indication for each treatment. The primary outcome measure was the target cost according to a production cost calculation algorithm. The secondary outcome measure was the target cost as the lowest available generic price; this was necessary where export data were not available to generate an estimate from our cost calculation algorithm. Other outcomes included patent expiry dates and total eligible treatment populations.

**Results:**

Target prices were £411 per cycle for bortezomib, £9 per month for dasatinib, £852 per month for everolimus and £10 per month for gefitinib. Compared with current list prices in England, these target prices would represent reductions of 74–99.6%. Patent expiry dates were bortezomib 2014–22, dasatinib 2020–26, everolimus 2019–25 and gefitinib 2017. The total global eligible treatment population in 1 year is 769 736.

**Conclusions:**

Our findings demonstrate that affordable drug treatment costs are possible for novel cancer drugs, suggesting that new therapeutic options can be made available to patients and doctors worldwide. Assessing treatment cost estimations alongside cost-effectiveness evaluations is an important area of future research.

**Strengths and limitations of this study**

- A conservative and inefficient manufacturing model was used to generate realistic target prices. Generic prices represent real-world market costs, which are likely to decrease in the future.
- We used peer-reviewed, publicly available epidemiological data to generate robust eligible treatment populations.
- The estimated treatment costs assume the absence of intellectual property monopolies which, for drugs under patent protection, may not be possible for several years.
- This study calculates realistic target treatment costs. Assessing the impact of target costs on cost-effectiveness, however, was beyond the scope of the present study.

## Introduction

In 2013, there were 8.3 million cancer deaths worldwide, representing 15% of overall mortality.^1^ There were an estimated 14 million incident cases in 2012, a figure that is expected to rise to almost 24 million by 2035.^2^ Most diagnoses occur in low and middle-income countries (LMICs). In 2009, the worldwide cost of incident cancer cases alone was estimated to be $286 billion.^3^ Over the past decade, several new classes of cancer drugs have entered markets across the world.^4^

The high prices of new cancer treatments are known to be a barrier to access in LMICs, where monthly drug prices often exceed annual incomes.^5^ These prices have begun to pose problems in high-income settings too: newer drugs are a major contributor to the 10-fold increase in the average cost of cancer treatment in the UK since 1995.^6^ Drug prices account for roughly a quarter of all cancer costs and prices have increased 10 times in the past decade.^7^ Price is a key factor behind disparities in cancer healthcare in Europe, where €13.6 billion was spent on cancer drugs in 2009, amounting to 27% of all cancer care costs.^8^ ^9^

While cancer medication costs continue to rise, there is only a weak correlation with improvements in clinical efficacy.^10^ The UK's National Institute for Health and Care Excellence (NICE) has on numerous occasions in recent years found new cancer medicines to be cost-ineffective compared with current standards of care, often because the significantly higher costs are not matched by an improvement in clinical efficacy of the same magnitude. Since 2000, 31% of all technology appraisals conducted by NICE for cancer drugs received the verdict ‘not recommended’, double the average for all treatments.^11^ For cancer medications, National Health Service (NHS) England has responded to accusations of ‘rationing’ by creating the controversial Cancer Drugs Fund(CDF).^12^ The CDF provides funding for drugs that have not received approval from NICE.

Recent analyses of the costs of production for hepatitis B and C medicines have prompted informed debate on the optimal provision of treatments and services within a constrained budget.^13^ ^14^ This study aims to provide similar analyses for clinical indications for novel cancer medicines that have been deemed cost-ineffective. We have analysed the potential impact of generic importation for four drugs, three of which (bortezomib, dasatinib and everolimus) have been deemed cost-ineffective by NICE, and are currently included on the CDF list.^15^

## Methods

### Calculation of production cost

Data on active pharmaceutical ingredients (API) exported from India were extracted from an online database for 2014 and early 2015.^16^ Given that prices of API decrease with continued market competition, we used the lowest per-kilogram API price in this timeframe in our calculations to estimate sustainable generic prices in the near future.

Per-kilogram API prices were input into an algorithm previously used in analyses of drugs for hepatitis B, C, and oncology drugs.^13^ ^14^

**Figure 1:**
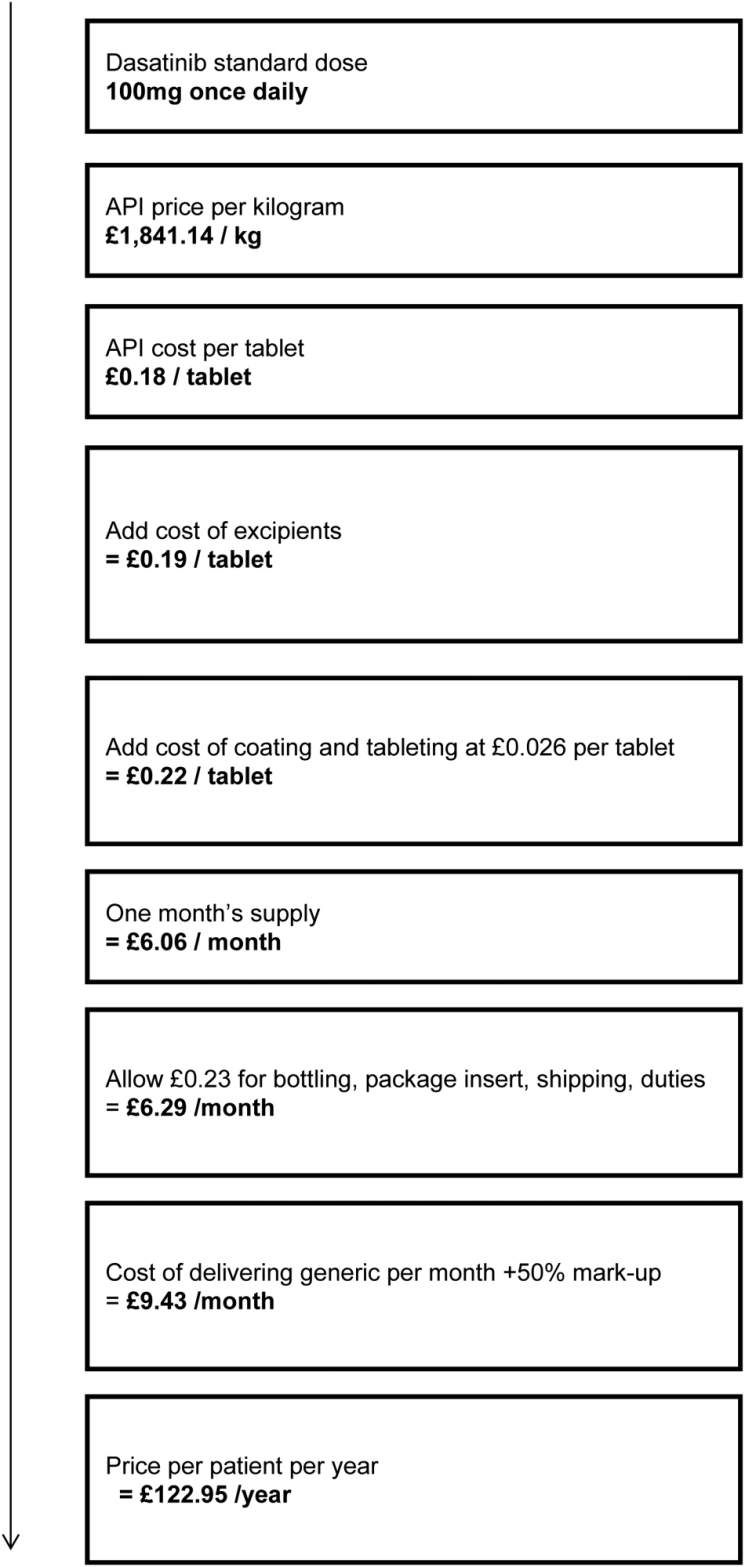
Cost estimation flow chart for dasatinib.

An example of our calculation algorithm for dasatinib is given in figure 1. The standard dose of dasatinib is 100 mg once daily. Thus, the yearly requirement of API is 36.5 g per patient. The lowest price for dasatinib API exported from India in 2014 was £1841.14/kg. The amount of API required to produce one 100 mg tablet would thus cost £0.18. The total weight of the tablet was assumed to be five times the weight of the API alone, and excipient prices were calculated by conservatively assuming that the total non-API mass of the tablet was composed of the most expensive excipient. The costs of excipients (£0.006 in the case of dasatinib, based on export data) and tableting (a conservative estimate of £0.026 per tablet) were added to the per-pill cost of the API. The resulting per-pill cost of production was multiplied by 28 to give the monthly cost of production (£6.06/month). Shipping costs and duties at £0.23 per month, assuming packaging in monthly quantities, were added giving a total monthly cost of £6.29. These assumptions are based on confidential contact with generic producers, and would reflect a relatively inefficient manufacturing process. Finally, a 50% mark-up was added, to include a profit margin that would incentivise market entry and competition between generic manufacturers, giving a final estimated generic price of £9.43/month, or £122.95 per patient per year.

### Patent coverage and global prices

**Figure 2:**
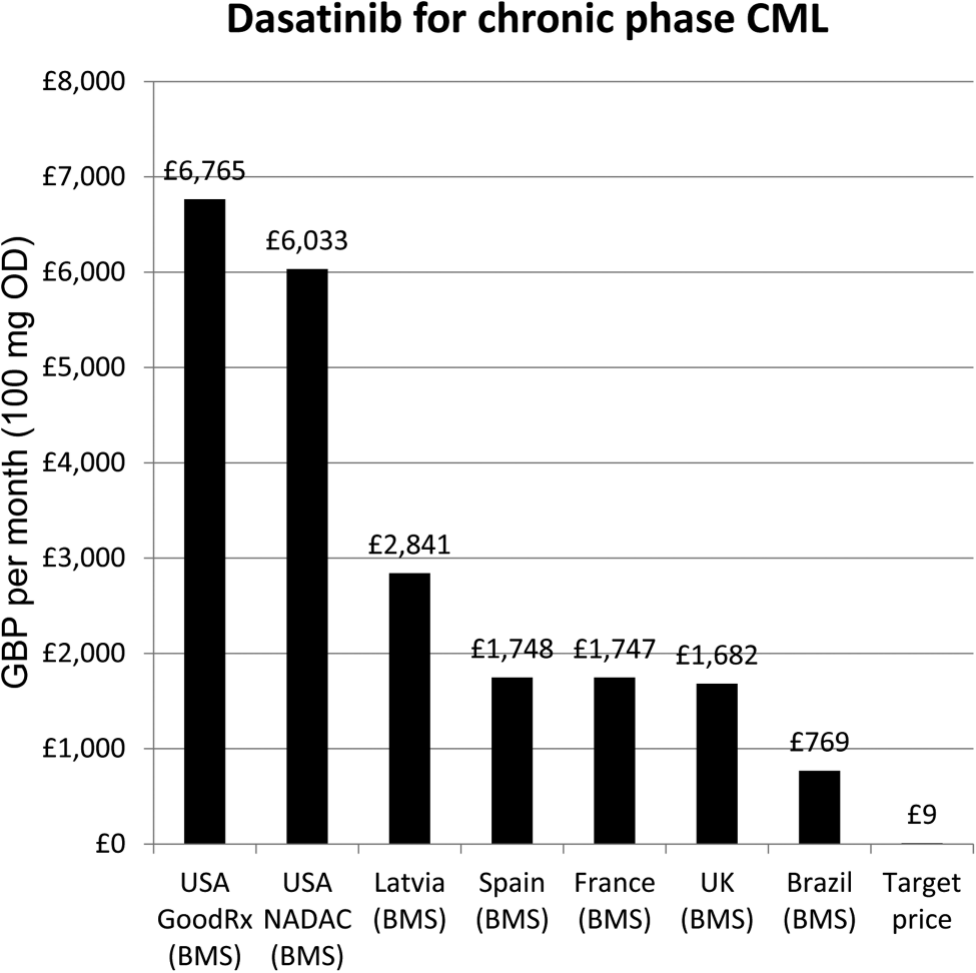
Lowest prices of dasatinib from selected countries.

US basic (substance) patent expiry dates were gathered from the Food and Drug Administration Orange Book.^17^ Prices for the chosen drugs were identified in eleven countries, using national databases and online price comparison tools (see online supplementary appendix A). In all cases, the lowest available price per pill was used for comparison. In cases where national pricing information was lacking, the corresponding bar is absent (figure 2).

10.1136/bmjopen-2016-011965.supp1 supplementary appendix

### Incidence of cancers and volume demand estimation

Using published figures of the epidemiology of cancers for which the chosen medicines are indicated, we estimated the annual volume of demand in terms of tonnes of API that would be required to treat all incident cases. We estimated the incidence of all cancers for which the four chosen drugs are indicated, including multiple myeloma, chronic myeloid leukaemia, acute lymphoblastic leukaemia, renal cell carcinoma and non-small cell lung cancer. The potential number of people newly eligible for treatment with each drug, per year, was multiplied by the annual requirement of API in grams per patient to give annual volume demand.

Incidence data for International Classification of Diseases, tenth edition (ICD-10) categories were obtained from *GLOBOCAN 2012*,^2^ and the incidence of specific cancer subtypes was estimated by combining these figures with published data from studies on the proportion of cases of the cancer subtype within the ICD-10 group. Estimates for the UK were developed using incidence data from the Cancer Research UK database. Taking chronic myeloid leukaemia as an example, it comprises 12.3% of the ICD-10 category ‘leukaemia’.^18^ For breast cancer, data were only available for women.^19^

The proportion of incident cases of cancer that would be eligible for treatment with each drug was calculated by using data on the prevalence of eligibility criteria such as the proportion with metastatic disease at presentation, or the proportion that are Philadelphia chromosome-positive.

As therapies for clear cell advanced/metastatic renal carcinoma are not curative, our analysis has assumed that all patients eligible for first-line treatment will progress and become eligible for second-line treatment with everolimus.^20^ For non-clear cell advanced/metastatic renal cell carcinoma, a consensus on which medicine is first-line has not yet emerged, with more than one medicine recommended as possible first-line agents. Dasatinib has been recommended as first-line treatment for Philadelphia chromosome-positive chronic myeloid leukaemia and Philadelphia chromosome-positive acute lymphoblastic leukaemia.^21^ ^22^ For the purposes of this analysis, all patients for whom everolimus and dasatinib are recommended as one of the possible first-line or second-line agents have been included in the eligible population; our estimates of numbers newly eligible for treatment with these drugs per year overlap, and would be affected by future changes in treatment guidelines.

Our estimates assumed full access to all interventions indicated before use of drugs, including surgery, radiotherapy and chemotherapy. We do not include measures of access in our assumptions; where patients do not have access to these interventions, drugs may provide the best available treatment due to low cost, potentially increasing the eligible population. In addition, data from high-income countries (HICs) for the proportion of cases that are advanced/metastatic at presentation are likely to underestimate the proportion in countries with reduced access to healthcare services and health information. Finally, our estimates use incidence data, thus giving the number *newly* eligible per year. The point prevalence of eligible people would by definition be greater.

## Results

### Calculated target prices

**Figure 3:**
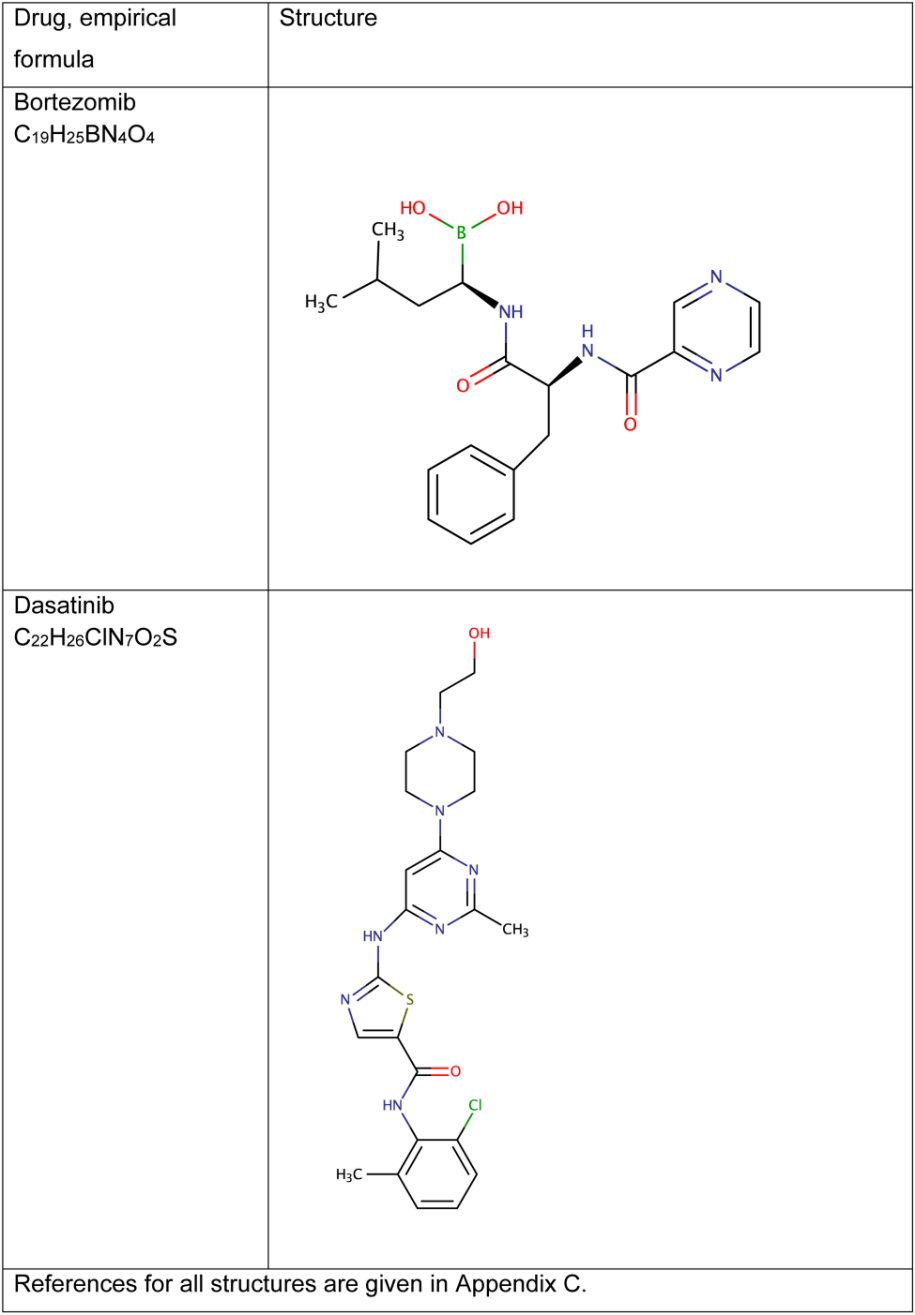
Chemical structures and formulas for bortezomib and dasatinib.

**Figure 4:**
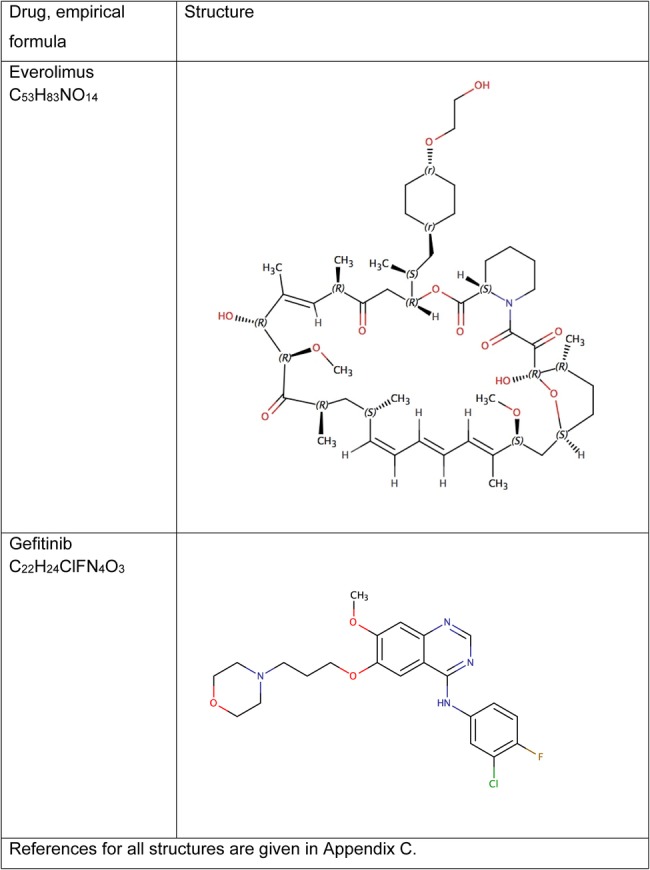
Chemical structures and formulas for everolimus and gefitinib.

Chemical structures are shown in figures 3 and 4, with references for these in online supplementary appendix B. API export data sufficient to allow calculation of generic price estimates were only available for dasatinib and gefitinib (table 1). For bortezomib and everolimus, the lowest priced product globally was used for comparisons with UK prices.

**Table 1:** Assumptions and calculations of target prices

Medicine	Dasatinib	Gefitinib
Daily dose	100 mg	250 mg
Tablets per month	28	28
API price per kilogram	£1841.14	£802.56
API cost per tablet	£0.18	£0.20
Add cost of excipients	£0.19	£0.21
Add cost of tableting	£0.22	£0.24
Cost per month	£6.06	£6.61
Add cost of bottle, packaging, shipping, duties	£6.29	£6.84
Add 50% mark-up	£9.43	£10.26
Target price per year	**£122.95**	**£133.73**

The prices of excipients used for each TKI are given in text, but not shown in table. Bold values are the final target price.

EGFR, Epidermal growth factor receptor; TKI, tyrosine kinase inhibitor.

10.1136/bmjopen-2016-011965.supp2 supplementary appendix

#### Bortezomib

The recommended dose for bortezomib is 1.3 mg/m^2^ for a body surface area of 1.8 m^2^, taken twice a week for two consecutive weeks, followed by a resting week, in a 3-week cycle. This is equivalent to a per-patient yearly API requirement of 159 mg.

**Figure 5:**
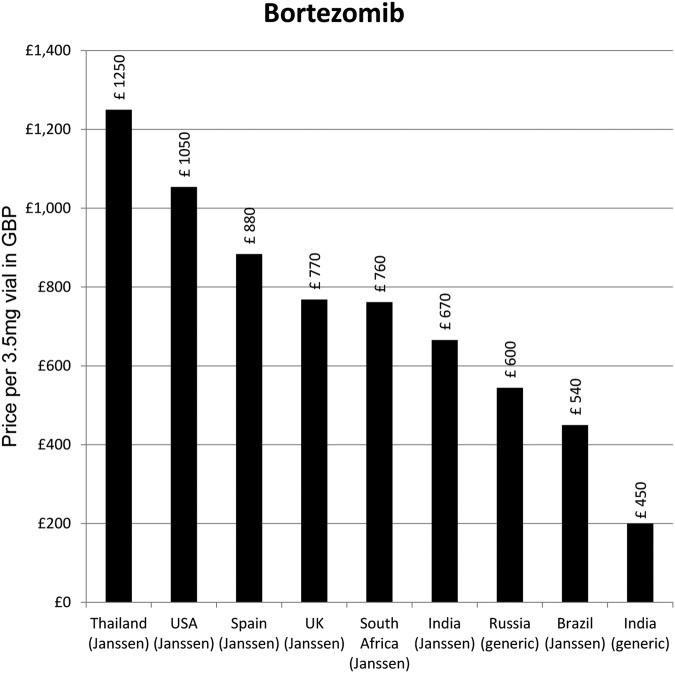
Lowest prices of bortezomib selected countries.

The lowest available generic price was for an Indian product: £199.92 per 3.5 mg phial (figure 5).

#### Dasatinib

The recommended dose for dasatinib is 100 mg taken once daily, equivalent to a per-patient yearly API requirement of 36.5 g.

17.5 kg of dasatinib API were exported from India in 2014–2015, with the largest volume shipment priced at £1841.14/kg. The most expensive excipient in dasatinib is hypromellose, costing £15.60/kg.

The estimated price for dasatinib, assuming a dose of 100 mg daily, was £122.95 GBP per year, or £9.43 GBP per month. The lowest available price was from the originator company in Brazil, costing £769.03 per month (figure 2).

#### Everolimus

**Figure 6:**
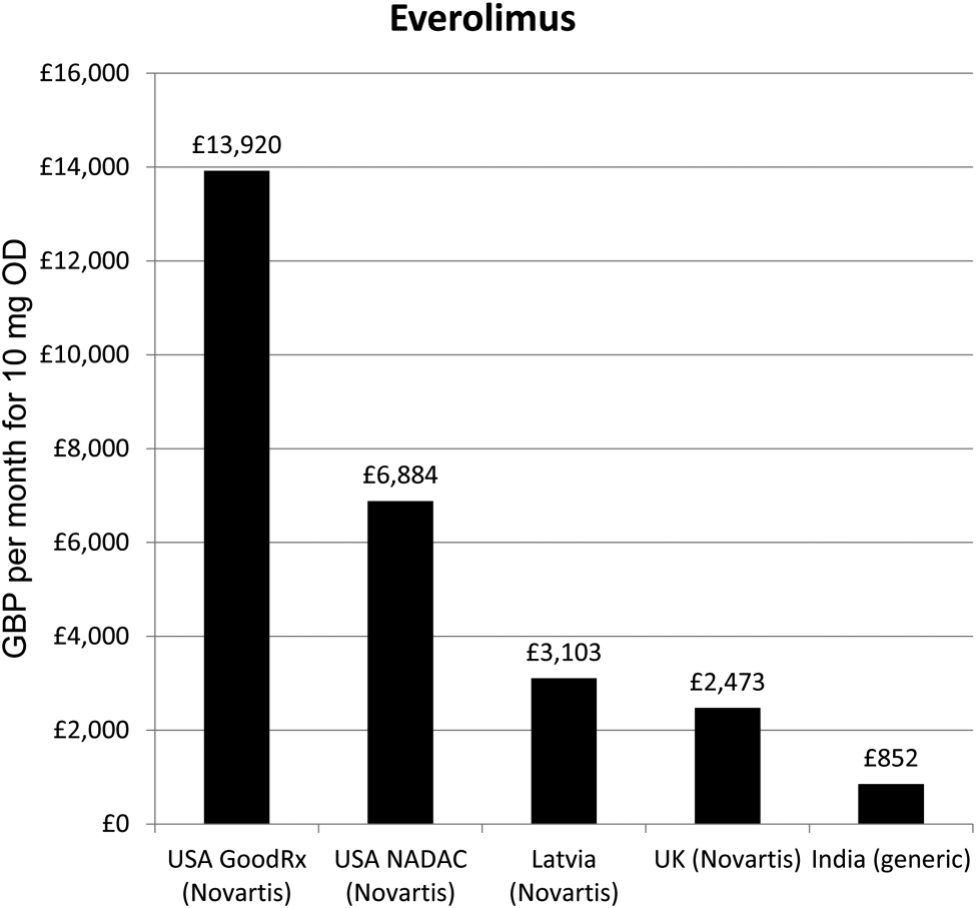
Lowest prices of everolimus from selected countries.

The recommended dose for everolimus is 10 mg daily, equivalent to a per-patient yearly API requirement of 3.7 g. The lowest available generic price globally was £688.96 per month, assuming off-label use, and £851.65 on-label, for Indian products (figure 6).

#### Gefitinib

The recommended dose for gefitinib is 250 mg once daily, equivalent to a per-patient yearly API requirement of 91.3 g. 416.8 kg of gefitinib API were exported from India in 2014–2015, with the largest single shipment priced at £802.56/kg. The most expensive excipient in gefitinib is povidone, costing £9.39/kg.

**Figure 7:**
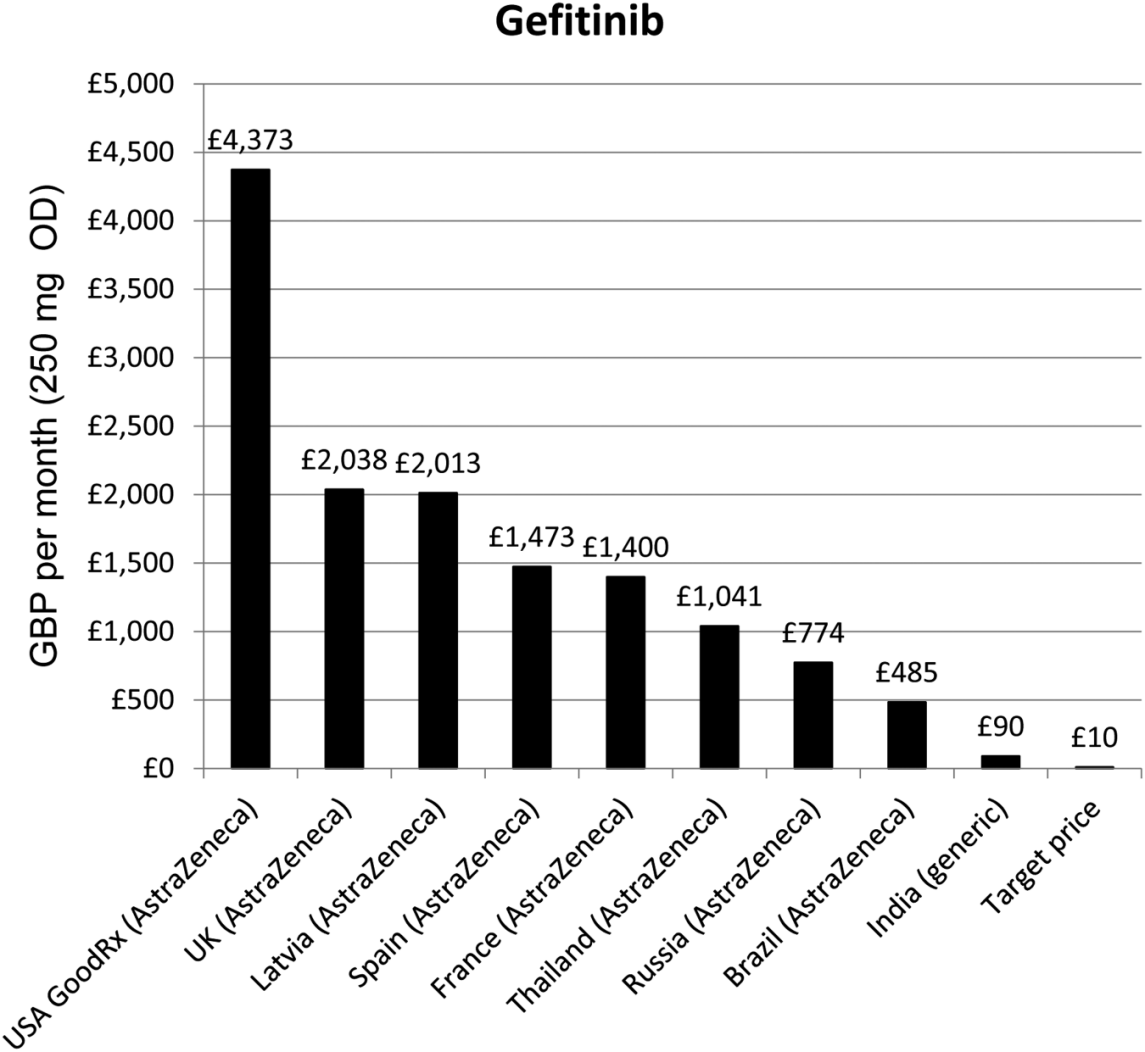
Lowest prices of gefitinib from selected countries.

The estimated price, assuming a daily dose of 250 mg, was £133.73 GBP per year, or £10.26 GBP per month. The lowest available generic price was £90.49 per month (figure 7).

### Patent expiry

Patent expiration dates for all drugs are shown in table 2. With the exception of bortezomib, for which the patent for one particular formulation of the drug expired in 2014, all drugs are currently under patent protection. Three of the drugs have multiple active patents, resulting in a range of expiration dates. Patent expiry dates were bortezomib 2014–22, dasatinib 2020–26, everolimus 2019–25 and gefitinib 2017.

**Table 2:** Current and target prices

Drug	Indication	Patent expiry	Current UK drug price per month (UK)*	Target price per month
Bortezomib^39^	1st line MM	2014–22	£762.38	£199.92
Dasatinib^40^	1st line CML	2020–26	£2504.96	£9.43
Dasatinib^41^	2nd line CML	2020–26	£2504.96	£9.43
Everolimus^42^	2nd line RCC	2019–25	£2970.00†	£851.65
Everolimus^43^	Breast CA	2019–25	£2970.00	£851.65
Gefitinib^44^	1st line NSC lung CA	2017	£2167.71†	£10.26

References for patent expiry dates in online supplementary appendix A.

*Monthly costs calculated using price from latest version of BNF Online.^45^

†A Patient Access Scheme (PAS) is in place for this drug. The PAS was not included in our calculations.

CA, cancer; CML, chronic myeloid leukaemia; MM, multiple myeloma; NSC, non-small cell; RCC, renal cell carcinoma.

### Global and UK demand

Incidence data and assumptions used to calculate eligible population estimates are presented in table 3 for the global population, and in table 4 for the UK population. References used are given in online supplementary appendix C.

**Table 3:** Global incidence of indicated cancers and estimates of total numbers eligible for treatment with selected medicine

Medicine	ICD-10 category and incidence	Indication of TKI, and percentage of relevant ICD-10 group	Eligibility in terms of pathology, and percentage of incident cases with this subtype	Eligibility in terms of stage of disease, a percentage of incident cases at this stage	Total number newly eligible for indication, per year	Total number eligible for drug, per year	Total API requirement per year
Bortezomib	Multiple myeloma, 114 251	–	–	Relapsed, received at least 1 prior therapy and who have already undergone or are unsuitable for haematopoietic stem cell transplantation, 25.5%	29 134	143 385	2.6 kg
	Multiple myeloma, 114 251	–	–	Patients for whom high-dose chemotherapy with stem cell transplantation is considered inappropriate, 86.4%	98 713		
	Multiple myeloma, 114 251	–	–	Patients for whom high-dose chemotherapy with stem cell transplantation is considered appropriate, 13.6%	15 538		
Dasatinib	Leukaemia, 351 965	Chronic myeloid leukaemia, 12.30%	Philadelphia chromosome-positive, 87.5%	Chronic phase, 90%	34 092	52 280	1.8 tonnes
	Leukaemia, 351 965	Chronic myeloid leukaemia, 12.30%	Philadelphia chromosome-positive, 87.5%	Intolerant or resistant to imatinib, 40%	15 152		
	Leukaemia, 351 965	Acute lymphoblastic leukaemia, 11.50%	Philadelphia chromosome-positive, 25%	Refractory to imatinib, 30%	3036		
Everolimus	Kidney, 337 860	Renal cell carcinoma, 85%	Clear cell renal cell carcinoma, 77.5%	Advanced/metastatic, 71.5%	159 134	282 678	1.0 tonnes
	Kidney, 337 860	Renal cell carcinoma, 85%	Non-clear cell renal cell carcinoma, 22.5%	Advanced/metastatic, 71.5%	46 200		
	Breast, 1 671 149	–	Advanced/metastatic, 29.5%	HER2 negative, post-aromatase inhibitor, 12.3%	60 638		
Gefitinib	Trachea, bronchus and lung (C33–34), 1 824 701	Non-small cell lung cancer, 85%	EGFR-positive, 22.5%	Advanced/metastatic, 83.5%	291 393	291 393	26.6 tonnes

Advanced pancreatic neuroendocrine and tuberous sclerosis, for which everolimus is an indicated treatment in some cases, has not been included, due to its relative rarity. Bortezomib is indicated in some cases of mantle cell lymphoma. This has not been included, due to lack of available data.

Dosages assumed: bortezomib—two cycles of 1.3 mg/m^2^ twice weekly for 2 weeks for body surface area of 1.73 m^2^, dasatinib—100 mg daily, everolimus—10 mg daily, gefitinib—250 mg daily.

API, active pharmaceutical ingredients; ICD-10, International Classification of Diseases, tenth edition

**Table 4:** UK incidence of indicated cancers, and estimates of total numbers eligible for treatment with selected medicine

Medicine	Incidence by ICD-10 category	Indication of medicine, and proportion of relevant ICD-10 group	Eligibility in terms of pathology, and percentage of incident cases with this subtype	Eligibility in terms of stage of disease, a percentage of incident cases at this stage	Total number eligible for indication, per year	Total number eligible for medicine, per year
Bortezomib	Multiple myeloma, 4792	–	–	Relapsed, received at least 1 prior therapy and who have already undergone or are unsuitable for haematopoietic stem cell transplantation, 25.5%	1222	6014
	Multiple myeloma, 4792	–	–	Patients for whom high-dose chemotherapy with stem cell transplantation is considered inappropriate, 86.4%	4140	
	Multiple myeloma, 4792	–	–	Patients for whom high-dose chemotherapy with stem cell transplantation is considered appropriate, 13.6%	652	
Dasatinib	Chronic myeloid leukaemia, 675	–	Philadelphia chromosome-positive, 87.5%	Chronic phase, 90%	532	817
	Chronic myeloid leukaemia, 675	–	Philadelphia chromosome-positive, 87.5%	Intolerant or resistant to imatinib, 40%	236	
	Acute lymphoblastic leukaemia, 654	–	Philadelphia chromosome-positive, 25%	Refractory to imatinib, 30%	49	
Everolimus	Kidney, 10 144	Renal cell carcinoma, 85%	Clear cell renal cell carcinoma, 77.5%	Advanced/metastatic, 71.5%	6165	9780
	Kidney, 10 144	Renal cell carcinoma, 85%	Non-clear cell renal cell carcinoma, 22.5%	Advanced/metastatic, 71.5%	1790	
	Breast, 50 285	–	Advanced/metastatic, 29.5%	HER2 negative, postaromatase inhibitor, 12.3%	1825	
Gefitinib	Lung cancer, 44 488	Non-small cell lung cancer, 85%	EGFR positive, 22.5%	Advanced/metastatic, 83.5%	7104	7104

Advanced pancreatic neuroendocrine and tuberous sclerosis, for which everolimus is an indicated treatment in some cases, has not been included, due to its relative rarity. Bortezomib is indicated in some cases of mantle cell lymphoma. This has not been included due to lack of available data.

10.1136/bmjopen-2016-011965.supp3 supplementary appendix

## Discussion

Significant price reductions can be achieved for numerous new cancer medicines, making new treatments available for an estimated 16 611 people in the UK each year, for those who live in England, these treatments are not currently funded by NHS England.

Generic production could allow the UK price of dasatinib to decrease by 99.6%, and the UK price of gefitinib to decrease by 99.5%. Importation of Indian generics would represent a UK price decrease of 74% for bortezomib and 71% for everolimus. No generic versions of dasatinib were identified in the countries surveyed. Generic versions of bortezomib were found in India and Russia. Generic everolimus was found in India. Generic gefitinib was found to be available only in India, for £90 per month. While this price is significantly below than that in other countries (figure 7), it is ninefold the estimated generic price of £10 per month. The current generic price of gefitinib in India is roughly equal, per year, to the median per annum income. It is therefore likely that the mark-ups set by the generic companies currently producing gefitinib are set with marketing to a wealthy subset of the Indian population in mind. A low volume of demand for gefitinib in India, due to, for example, limited state cancer treatment programmes, may also be a contributing factor for the relatively high price.

We estimate that globally, there are 769 736 newly diagnosed patients with cancer every year that could be treated with one of these four drugs. Providing these drugs to all eligible patients, at target prices, would cost an estimated £2.9 billion.

The target prices presented in this paper are based on real-world export and pricing data, calculated using a conservative algorithm that assumes a relatively inefficient manufacturing process and includes shipping and tableting costs, as well as a significant profit margin.

Our predictions assume market sizes of a volume sufficient to attract generic producers. For cancer drugs with smaller patient populations, reductions may be harder to achieve. Allowing for sufficient demand, and a permissive legal environment, our findings demonstrate realistic future prices for novel cancer drugs. The price reductions seen in HIV drugs over the past two decades show the dramatic effects of robust generic competition on access to medicines.^23^ While our estimates focus on chemically derived medicines, biologics represent a growing proportion of new cancer medications.^24^ The complex molecular structures of biologics present regulatory and manufacturing challenges to the production of low-cost off-patent biosimilars meaning that, so far, only price reductions of between 10% and 35% have been achieved.^25^ While it may not be possible to achieve the same level of reductions as seen in generics, it is likely that, as manufacturing and regulatory processes mature, and clinicians and patients become more familiar with biosimilars, the size of price reductions will increase in the future.^25^

Patent expiry dates for the medicines included in this study range from 2014 to 2026. For bortezomib and gefitinib, generic competition is likely to be possible in the next few years, whereas for everolimus and dasatinib, patent protection is likely to prevent the competition necessary to reach the target prices. The time to generic market entry from patent expiry varies significantly between countries. Hudson analysed generic entry between 1985 and 1996, finding a range in average time to entry of between 1.26 and 3.4 years; however, for a sample of generics licensed in the EU between 2000 and 2007, this ranged from 4 to 7 months, suggesting entry-lag times are decreasing.^26^ ^27^ There are numerous strategies that high, low and middle-income countries can use to decrease entry-lag. These include supply-side policies such as expedited drug approval processes, and demand-side policies such as pricing policies.^28^ ^29^

Several options exist for national governments wishing to facilitate access to medicines by altering the patent status. Compulsory License (CL) legislation permits a state to license a patented drug without the patent holder's consent. Although their use is infrequent, CLs are an effective method of facilitating generic competition, provided for under international agreements signed by all 161 member countries of the World Trade Organisation (WTO).^30^ A CL can only be granted after a state has made meaningful efforts to negotiate a price, unless there is a state of national emergency or ‘extreme urgency’, conditions that the state can determine for itself, in which case the state may proceed directly to a CL. Importantly, the patent holder must still receive reasonable remuneration for the CL.^31^ The WHO has published guidelines on remuneration of patent holders which may help facilitate the pursuit of non-voluntary licences.^32^ Relevant domestic legislation may also provide a useful method of negating the barriers posed by patents, because they may provide for different conditions to those legislated by the WTO's Trade Related Aspects of Intellectual Property rights (TRIPS) agreement. In the UK, Crown Use provisions allow the government to use or license a patent in the name of the public good, and are currently being considered for use with the monoclonal antibody conjugate, trastuzumab emtansine for refractory breast cancer.^33^ ^34^ Only dasatinib, of the drugs included in our study, has been the subject of CL efforts.^35^ Even if they are ultimately not realised, the CL approach may bring price reductions as originator companies respond to a change in negotiations.

In some cases, voluntary licenses can be agreed between originator companies and interested third parties, facilitating generic production under the terms of license. This approach has most notably been used with HIV drugs due to the work of Medicines Patent Pool, although it was also used for Gilead Sciences’ breakthrough hepatitis C drug, sofosbuvir.^36^ ^37^

In other cases, patents may be challenged outright. Section 3(d) of the Indian Patent Act allows third parties to challenge patent validity, which has in the past led to the revocation of patents on cancer drugs, and consequent generic production.^38^ While it is beyond the scope of this paper to discuss whether these drugs are suitable candidates for such an approach, it is notable that dasatinib has been at the centre of a patent dispute in India.

## Conclusion

Using real-word export data and a conservative manufacturing model, we calculated realistic target prices for four cancer drugs. We predict that the resulting price reductions would have a significant effect on their cost-effectiveness in six clinical indications, making them viable treatment options for more than 750 000 patients worldwide each year. Some of these clinical indications are currently deemed unaffordable by NICE using cost-effectiveness criteria, but if the realistic target price was available, all the drugs may satisfy NICE's criteria, removing the need for additional funding through initiatives such as the CDF.

Currently, the existing patents on the drugs are the major barrier to achieving predicted target prices, which rely on robust generic competition. Numerous strategies exist for the UK government to pursue in this regard, such as those suggested for the drug trastuzumab emtansine. In any case, knowledge of realistic treatment production costs will be beneficial to price negotiations across the world.
