# Supplementary material for: Estimated generic prices of cancer medicines deemed cost-ineffective in England: a cost estimation analysis
Source: BMJ Open. 2017 Jan 20;7(1):e011965. doi: 10.1136/bmjopen-2016-011965 (PMC5253524; doi:10.1136/bmjopen-2016-011965)
Supplement: supplementary appendix [file bmjopen-2016-011965supp_appendixA.pdf]

Appendix A- Data sources and references for drug prices

All prices were converted from national currency to USD using exchange rates given at http://www.xe.com/currencyconverter/ on the 13th of July 2015.

For Canada, prices in the province of Québec are used.

Country	Price source
USA	GoodRx. http://www.goodrx.com/.
South Africa	South African Medicine Price Registry. Database of Medicine Prices. http://www.mpr.gov.za/Publish/ViewDocument.aspx?DocumentPublicationId=1761.
Spain	Colegio de Farmaceuticos de Ponteverda. Consulta de Precios de Medicamentos. http://www.cofpo.org/index.php/medic-es.html?order_by=&sort=&per_page=35&search=descripcion&for=interferon.
UK	British National Formulary. https://www.medicinescomplete.com/mc/bnf/current/.
France	Ministère des Affairs sociales et de la Santé. Recherche Par Medicament. http://medicprix.sante.gouv.fr/medicprix/rechercheSpecialite.do?parameter=rechercheSpecialite.
Thailand	Drug And Medical Supply Information Center. Ministey of Public Health. http://dmsic.moph.go.th/.
Russia	Государственный реестр предельных отпускных цен. http://grls.rosminzdrav.ru/PriceLims.aspx.
Canada	Régie de l’assurance maladie du Québec. List of Medications. http://www.ramq.gouv.qc.ca/en/regie/legal-publications/Pages/list-medications.aspx.
Brazil	Transparência Pública. Licitações - Advanced search. http://www3.transparencia.gov.br/TransparenciaPublica/jsp/licitacoes/licitacaoBuscaAvancada.jsf?consulta2=5&camposDefault=true&CodigoOrgao=null.
Latvia	Zāļu valsts aģentūra. Zāļu cenu pārbaudes forma. http://www.zva.gov.lv/?id=588&top=588&sa=111.
India	DrugsUpdate.com. http://www.drugsupdate.com/.
