# Supplementary material for: Estimated generic prices of cancer medicines deemed cost-ineffective in England: a cost estimation analysis
Source: BMJ Open. 2017 Jan 20;7(1):e011965. doi: 10.1136/bmjopen-2016-011965 (PMC5253524; doi:10.1136/bmjopen-2016-011965)
Supplement: supplementary appendix [file bmjopen-2016-011965supp_appendixB.pdf]

Appendix B- References for the chemical structures of each drug

Bortezomib

Royal Society of Chemistry, 2015. Bortezomib. *ChemSpider*. Available at: http://www.chemspider.com/Chemical-Structure.343402.html[Accessed August 10, 2015].

National Centre for Biotechnology Information, 2015. Bortezomib. *PubChem*. Available at: http://pubchem.ncbi.nlm.nih.gov/compound/Bortezomib [Accessed August 10, 2015].

Dasatinib

National Centre for Biotechnology Information, 2015. Dasatinib. *PubChem*. Available at: http://pubchem.ncbi.nlm.nih.gov/compound/Dasatinib#section=Top [Accessed August 10, 2015].

Everolimus

National Centre for Biotechnology Information, 2015. Everolimus. *PubChem*. Available at: http://pubchem.ncbi.nlm.nih.gov/compound/Everolimus#section=Top [Accessed August 10, 2015]

Gefitinib

National Centre for Biotechnology Information, 2015. Gefitinib. *PubChem*. Available at: http://pubchem.ncbi.nlm.nih.gov/compound/Gefitinib [Accessed August 10, 2015].
