# Supplementary material for: Estimated generic prices of cancer medicines deemed cost-ineffective in England: a cost estimation analysis
Source: BMJ Open. 2017 Jan 20;7(1):e011965. doi: 10.1136/bmjopen-2016-011965 (PMC5253524; doi:10.1136/bmjopen-2016-011965)
Supplement: supplementary appendix [file bmjopen-2016-011965supp_appendixC.pdf]

Appendix C- Methodology and references for eligible treatment populations

Renal cell carcinoma

85% of kidney cancers[1]

Clear cell carcinoma – 75-80% of kidney cancer. Average 77.5%

Nonclear cell carcinoma – 20-25% of kidney cancer. Average 22.5%

Advanced/metastatic – 71.5%[2] [NICE guidance states 26% and 17% have stage III and IV disease, and about half of those with curative resection for earlier stages of the disease also go on to develop advanced and/or metastatic disease. Calculation 26+17+(0.5x57) = 71.5%]

Breast cancer

Metastatic breast cancer at presentation 5%, with 35% who present with local breast cancer who will progress. Total 38.25%[3]

20-30% with metastatic breast cancer are HER2+, of which 50% will also be hormone receptor positive[4]

Average 12.5%

Chronic Myeloid Leukaemia

12.3% of Leukaemia (C91-95)[5]

Philadelphia chromosome positive 85-90%[6]

Acute Lymphoblastic Leukaemia

11.5% of Leukaemia (C91-95)[5]

Philadelphia chromosome positive 25%[7]

References

1. Weikert S, Ljungberg B. Contemporary epidemiology of renal cell carcinoma: perspectives of primary prevention. [cited 2015 Mar 19];28(3):247–52. Available from: http://search.proquest.com/docview/220282324/abstract?accountid=14511

2. Sunitinib for the first-line treatment of advanced and/or metastatic renal cell carcinoma | 2-clinical-need-and-practice | Guidance and guidelines | NICE. NICE; [cited 2015 Mar 29]; Available from: https://www.nice.org.uk/guidance/ta169/chapter/2-clinical-need-and-practice

3. National Institution of Clinical Excellence. Everolimus (Afinitor) in combination with exemestane for the treatment of advanced or metastatic HER2 negative, hormone receptor positive breast cancer after prior endocrine therapy. Single technology appraisal (STA) [Internet]. 2012. Available from: http://www.nice.org.uk/guidance/ta295/documents/breast-cancer-her2-negative-

oestrogen-receptor-positive-locally-advanced-or-metastatic-everolimus-with-an-aromatase-inhibitor-afinitor2

4. Doss S, Robertson J, Adam J. Lapatinib or trastuzumab in combination with an aromatase inhibitor for first-line treatment of metastatic hormone-receptor-positive breast cancer that overexpresses HER2. Lancet Oncol. 2012;13(September 2009):766–7.

5. American Cancer Society. Cancer Facts & Figures 2015. Atlanta: American Cancer Society; 2015.

6. Demiroglu A, Steer EJ, Heath C, Taylor K, Bentley M, Allen SL, et al. The t(8;22) in chronic myeloid leukemia fuses BCR to FGFR1: transforming activity and specific inhibition of FGFR1 fusion proteins. Blood [Internet]. 2001 Dec 15 [cited 2015 Mar 29];98(13):3778–83. Available from: http://www.ncbi.nlm.nih.gov/pubmed/11739186

7. Moorman A V, Harrison CJ, Buck GAN, Richards SM, Secker-Walker LM, Martineau M, et al. Karyotype is an independent prognostic factor in adult acute lymphoblastic leukemia (ALL): analysis of cytogenetic data from patients treated on the Medical Research Council (MRC) UKALLXII/Eastern Cooperative Oncology Group (ECOG) 2993 trial. Blood [Internet]. American Society of Hematology; 2007 Apr 15 [cited 2015 Mar 18];109(8):3189–97. Available from: http://www.bloodjournal.org/content/109/8/3189.abstract
